# Exploring Edible Mushrooms for Diabetes: Unveiling Their Role in Prevention and Treatment

**DOI:** 10.3390/molecules28062837

**Published:** 2023-03-21

**Authors:** Mohammad Zaki Shamim, Awdhesh Kumar Mishra, Tahreem Kausar, Saurov Mahanta, Bhaskar Sarma, Vijay Kumar, Piyush Kumar Mishra, Jibanjyoti Panda, Kwang-Hyun Baek, Yugal Kishore Mohanta

**Affiliations:** 1 Department of Food Nutrition and Dietetics, Faculty of Sciences, Assam Down Town University, Guwahati 781026, Assam, India; 2 Department of Biotechnology, Yeungnam University, Gyeongsan 38541, Republic of Korea; 3 Department of Food Technology, School of Interdisciplinary Sciences and Technology, Jamia Hamdard, Hamdard Nagar, New Delhi 110062, Delhi, India; 4 Guwahati Centre, National Institute of Electronics and Information Technology (NIELIT), Guwahati 781008, Assam, India; 5 Department of Botany, Dhemaji College, Dhemaji 787057, Assam, India; 6 Department of Orthopedics Surgery, Johns Hopkins University School of Medicine, Baltimore, MD 21205, USA; 7 Department of Botany, B. N. College, Dhubri 783324, Assam, India; 8 Department of Applied Biology, School of Biological Sciences, University of Science and Technology Meghalaya (USTM), Techno City, 9th Mile, Baridua, Ri-Bhoi 793101, Meghalaya, India

**Keywords:** edible mushroom, polysaccharides, diabetes mellitus, hyperglycemia, dietary supplement

## Abstract

Diabetes mellitus is a complex illness in which the body does not create enough insulin to control blood glucose levels. Worldwide, this disease is life-threatening and requires low-cost, side-effect-free medicine. Due to adverse effects, many synthetic hypoglycemic medications for diabetes fail. Mushrooms are known to contain natural bioactive components that may be anti-diabetic; thus, scientists are now targeting them. Mushroom extracts, which improve immune function and fight cancer, are becoming more popular. Mushroom-derived functional foods and dietary supplements can delay the onset of potentially fatal diseases and help treat pre-existing conditions, which leads to the successful prevention and treatment of type 2 diabetes, which is restricted to the breakdown of complex polysaccharides by pancreatic-amylase and the suppression of intestinal-glucosidase. Many mushroom species are particularly helpful in lowering blood glucose levels and alleviating diabetes symptoms. Hypoglycaemic effects have been observed in investigations on *Agaricussu brufescens*, *Agaricus bisporus*, *Cordyceps sinensis*, *Inonotus obliqus*, *Coprinus comatus*, *Ganoderma lucidum*, *Phellinus linteus*, *Pleurotus* spp., *Poria cocos*, and *Sparassis crispa*. For diabetics, edible mushrooms are high in protein, vitamins, and minerals and low in fat and cholesterol. The study found that bioactive metabolites isolated from mushrooms, such as polysaccharides, proteins, dietary fibers, and many pharmacologically active compounds, as well as solvent extracts of mushrooms with unknown metabolites, have anti-diabetic potential in vivo and in vitro, though few are in clinical trials.

## 1. Introduction

Human life expectancy rose to 50–60 years by the beginning of the twentieth century because of better medical, food, and sanitary conditions. However, it is predicted that by the twenty-first century, civilization will have a life expectancy of 80–90 years [1]. Nonetheless, the emergence of chronic inflammation and a reduction of immunological competence due to age contribute to neurological disorders, such as Alzheimer’s disease, dementia, Parkinson’s disease, atherosclerosis, diabetes, stroke, sarcopenia, and cancer [2].

Diabetes mellitus is a chronic metabolic disorder characterized by an excessive increase in glucose levels in the serum as a result of unbalanced insulin production and/or acuity to the hormone’s action on cellular receptor signaling. Modifications in glucose, lipid, and protein metabolism encompass these metabolic alterations. Hyperglycemia is the principal cause of most type 2 diabetes problems in individuals [3,4]. The suppression of complex polysaccharide breakdown by pancreatic α-amylase and limiting glucose absorption by blocking the intestinal α-glucosidase enzyme are two efficient techniques for type 2 diabetes treatment. Commercial medications for α-glucosidase inhibition in the treatment of diabetic illness include acarbose, miglitol, and voglibose [5]. Chronic inhibition of α-amylase and α-glucosidase may be beneficial in the treatment of type 2 diabetes and obesity [6,7]. As reversible inhibitors of α-glucosidase and α-amylase, the medications that are currently used to treat diabetic patients have adverse effects such as gastrointestinal disorders, meteorism, gas, bloating, and possibly even diarrhea [8].

Mushrooms have been used as food and medicine for a wide range of conditions for centuries. Although research is still in its infancy, culinary-medicinal mushrooms have shown promise as a potential treatment for a range of degenerative neurological disorders associated with aging, such as Parkinson’s and Alzheimer’s diseases [9]. Since ancient times, mushrooms have been included in the regular diets of many different cultures as a supplementary food item that is also high in nutrition [10]. Mushroom extracts contain fibers, proteins, polyphenols, lectins, alkaloids, and polysaccharides, all of which enhance their effectiveness [11,12,13]. As a result of clinical investigations, mushrooms have already proven themselves as a possible source of medications for both communicable and noncommunicable disorders. They also include a variety of unique secondary metabolites, polysaccharides, vital minerals, proteins, and vitamins, which supplement the primary diet in routine life [14,15,16]. Yet, only 10% of the world’s current mushroom species have been described by scientists, and just 1% of them have been used for therapeutic purposes. In this context, mushrooms appear to be a natural resource that has been underutilized to a significant extent, and their potential therapeutic use deserves scientific inquiry in the pursuit of new drugs [17].

It is believed that mushrooms possess qualities that protect against cancer, genotoxicity, oxidative stress, inflammation, high cholesterol levels, platelet aggregation, high blood pressure, immunological suppression, high glucose levels, and bacterial growth [18,19,20]. As both a functional food and a dietary supplement, mushrooms have the potential to help in the treatment of pre-existing health issues and the prevention of the development of potentially fatal [21] diseases. The nutritional and/or medicinal benefits of mushrooms, as well as their pharmacological effects, are improved by a balanced diet that includes mushrooms [10]. The term “mushroom” denotes the edible and medicinal fruiting body of a higher fungus.

Medicinal mushrooms and their active ingredients, including polysaccharides and their protein complexes, dietary fiber, and other compounds extracted from cultured mycelium, fruiting bodies, or broth, have been reported to have anti-hyperglycemic activity. These compounds exhibit anti-diabetic activity through various mechanisms [22]. Multiple mechanisms exist, both insulin-dependent and insulin-independent, in medicinal mushrooms for glucose control in diabetes, such as the inhibition of glucose absorption, increased insulin release, protection from beta-cell damage, improved antioxidant defenses, modulation of carbohydrate pathways, and reduced inflammation [23].

The main parts of the global mushroom industry are edible mushrooms, wild mushrooms, and medicinal mushrooms. The production of cultivated and edible mushrooms has increased more than 30-fold in the world since 1978. China is the leading producer of cultivated, edible mushrooms. *Lentinus edodes* is now the world’s leading cultivated edible mushroom, contributing to about 22% of the world’s supply. *Lentinula* and four other genera of mushrooms (*Pleurotus*, *Auricularia*, *Agaricus*, and *Flammulina*) account for 85% of the world’s total supply of cultivated edible mushrooms. On average, consumers now have access to about 5 kg of mushrooms per person per year. It is anticipated that mushroom consumption will increase on a per capita basis as more people become aware of the numerous health benefits associated with including mushrooms in their diet [24].

More and more research suggests that postmenopausal women who take anti-diabetic medications face an increased risk of fractures. It is anticipated that both longer drug use and higher dosages contribute to this risk. There is an increased risk of cardiovascular complications when taking anti-diabetic medicines, and these drugs also have hepatotoxicity. One of the most typical and severe diabetic consequences is nephropathy. A recent study suggests that there is a multifaceted interaction between the kidney and anti-diabetic drugs [25].

Wild edible mushrooms have higher levels of protein and vitamins, such as B vitamins, vitamin D, vitamin K, and, in some cases, vitamins A and C, as they possess unique natural chemical along with nutritional properties [26]. Moreover, mushrooms are low in fat and high in dietary fiber, nutraceuticals, and polysaccharides, all of which have been linked to improved health outcomes for a number of disorders. In addition to homoglucans (β-1, 3 glucan), heteroglycans, heterogalactans, and heteromannans, mushrooms also contain other structural polysaccharides. By inhibiting glucosidase, supporting the functions of glucose transporter 4, and lowering inflammatory markers, these polysaccharides and terpenoids (secondary metabolites) play a crucial role in glucose homeostasis and enhance insulin resistance and lipid metabolism [27,28].

**Figure 1:**
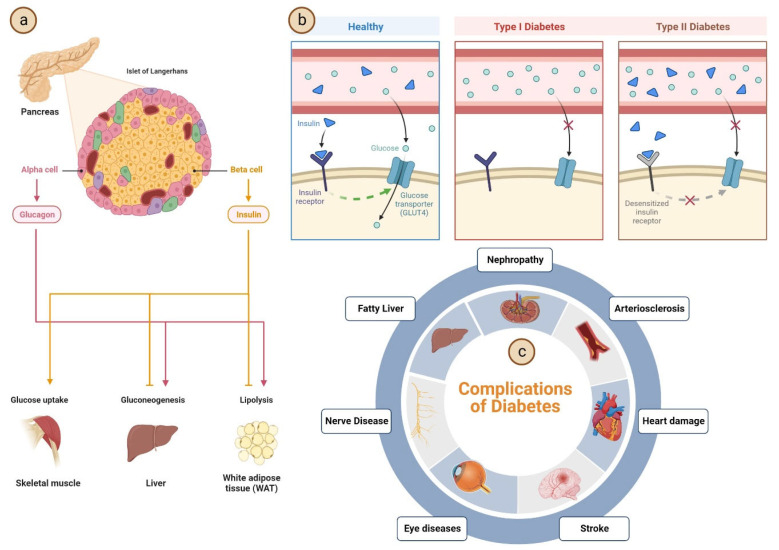
(**a**) Role of pancreases in glucose metabolism and maintenance of diabetes. (**b**) Comparative mechanism of different types of diabetes in the human body. (**c**) Different consequences of diabetes to the other organs and cellular systems.

The primary objective of this article is to make clear the function of mushrooms in the treatment of diabetes by investigating the potential bioactive compounds they contain, the pathophysiology of insulin resistance (IR), and the preventive mechanism of IR that can be accomplished with the help of mushrooms (Figure 1). In addition to this, some highlights have been presented on the nutraceutical potential as well as a functional meal based on mushrooms for the prevention and treatment of diabetes.

## 2. Mushroom Production Worldwide and Consumption

**Figure 2:**
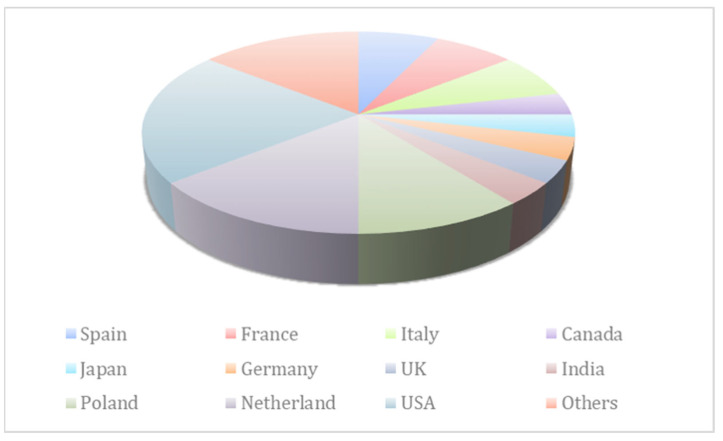
International comparative data on the production of mushrooms both wild as well as cultivated in these countries mentioned in the graphs and their most appropriate utility in food and medicine [29].

China, the United States of America, and the Netherlands are the three most important countries in terms of mushroom production; together, they are responsible for more than sixty percent of the total mushroom production around the globe. The whole mushroom production around the world is contributed to by China alone to the extent of 46 percent. The total amount of mushrooms produced in India comes to 1 lakh metric tons, which is equivalent to 3% of the world’s total production. China has the world’s highest consumption level per person, followed by the United States of America and other European nations, such as the Netherlands, Poland, France, and Spain (Figure 2).

## 3. Biological Activity of Mushrooms against Diabetes

**Figure 3:**
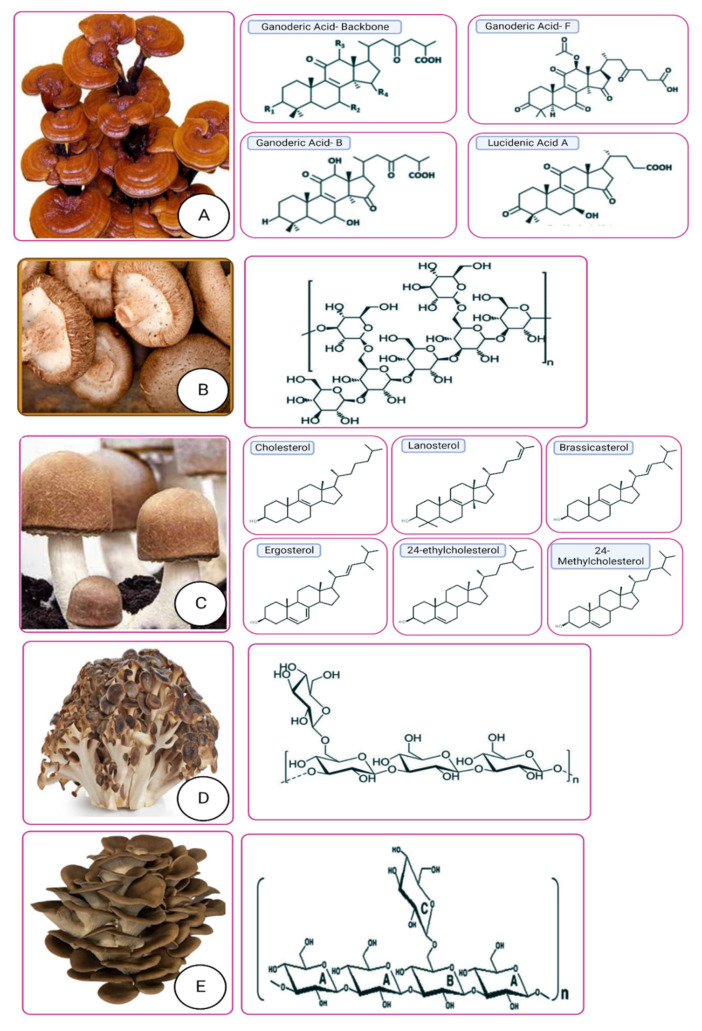
Bioactive compounds from mushrooms for potential nutraceuticals as well as medicinal uses: (**A**) *Ganoderma lucidum* (Lingzhi/Reishi); (**B**) *Lentinus edodes*; (**C**) *Agaricusblazeimurill*; (**D**) *Grifolafrondosa*; (**E**) *Pulmonariuspleurotus*.

Over 2000 edible mushrooms have been identified, with many of these mushrooms being consumed on a large scale due to their health benefits. The physiologically active substances, such as polysaccharides, produced throughout their development and proliferation are responsible for these health benefits. Homopolysaccharide glucan is one of the most physiologically active polysaccharides that are found in mushrooms. Mushroom fungal-glucans are polymers of D-glucose that are connected by (1, 3)-(1, 6) glycoside linkages. These bonds are hard to dissolve by pancreas-secreted enzymes [30]. It has been shown that polysaccharides derived from the mushrooms *Pleurotus ostreatus*, *Schizophyllum commune*, *Grifola rondosea*, *Sclerotium rolfsii*, *Ganoderma lucidum, Lentinus edodes,* and *Hericium erunaceus* have a therapeutic effect against metabolic syndrome, which is characterized by obesity, hypertension, and elevated blood sugar levels [31,32,33,34]. In the case of diabetes, the bioactive components (Figure 3) are significant in the analyses of anti-diabetic action, the level of increase in blood glucose, the scale of lipoproteins, the impact on immunity, the degree of insulin in the blood serum, the antioxidant consequence, the influence on the intestinal microbiota, fundamental changes in pancreas β-cells, and so on [30]. Some of the very frequently used and under high investigation of the nutraceutical developments worldwide.

### 3.1. Ganoderma lucidum (Lingzhi/Reishi)

It is a member of the Ganodermaceae family, which is well-known for its health benefits and lifespan. In China, it is known as ‘Ligzhi,’ while in Japan, it is known as ‘Reishi.’ Polysaccharides, β-glucans, organic germanium, phenols, lectins, steroids, lignins, mycins, amino acids, and vitamins are among its bioactive molecules [35,36]. Ganoderma is a gold-colored medicinal fungus [37]. *Ganoderma lucidum* is often mixed with *Cordyceps sinensis*, a fungus that is considered to boost *G. lucidum’s* efficacy [38].

### 3.2. Lentinus edodes (Shiitake Mushroom)

*Lentinus edodes* (Shiitake mushrooms) are frequently used as a treatment for the common cold, and this practice has a history that goes back hundreds of years. *L. edodes* is rich in a variety of important nutrients, including fiber, β-glucans, proteins, lipids, carbohydrates, minerals, vitamins B1, B2, and C, ergosterol, lectins, and lentinans [39]. Studies have shown that shiitake mushrooms can help protect pancreatic beta cells, boost insulin production, and lower blood glucose levels. When compared to normal rats, diabetic rats that had been streptozotocin-induced responded to an ex-polymer of *L. edodes* with decreased plasma glucose levels and increased insulin production [40].

### 3.3. Ophiocordyceps sinensis (Caterpillar fungus)

It was formerly known as *Cordyceps sinensis* and has been utilized as a medicinal fungus throughout history. It can only be found growing at high elevations in the Qinghai-Tibetan plateau, and it has become one of the most costly mushrooms used in eastern medicine due to high demand and declining yield. This mushroom can only be found in the presence of insect larvae, and it requires a rarefied atmosphere, mineral-rich soil, and low temperatures, making artificial production difficult. *Cordyceps* has a unique secondary metabolite profile, making it the world’s most expensive medicinal fungus [40]. *Cordyceps sinensis* has been proven to have hyperglycemic, anti-hypertensive, antioxidant, and cholesterol-lowering properties in animal, anti-atherosclerotic, and human trials [38].

### 3.4. Agaricus blazeimurill

*Agaricus blazeimurill* is native to Brazil but is also frequently cultivated in Japan. Historically, it was believed that this mushroom could reduce both physical and mental stress, boost the immune system, and assist in the management of diabetes, high cholesterol, and digestive issues. Ergosterols, lignins, and polysaccharides are the primary components that are responsible for its bioactivity. [41]. From both in vitro and in vivo investigations, polysaccharides, α-glucans, and β-glucans found in *A. blazeimurill* have been explored regarding their immune-modulatory and anti-mutagenic effects [40]. The polysaccharides present in this mushroom have been demonstrated to be useful in managing diabetes through processes such as (a) increasing plasma insulin and decreasing pancreatic glucagon; (b) Increasing insulin sensitivity and improving insulin resistance; (c) inhibiting the alpha-glucosidase enzyme; (d) increasing hepatic glycogen and sugar dysplasia; (e) increasing gluconeogenesis in peripheral tissues; (f) free radical scavenging and lipid peroxidation [42].

### 3.5. Grifola frondosa

It is often known as Maitake mushrooms and is popular in Korea, China, and Japan for its therapeutic benefits. Chinese people have consumed Maitake mushrooms for hundreds of years due to their delectable flavour. These mushrooms have anti-diabetic, immune-regulating, anti-tumor, and anti-hepatitis/HIV/AIDS properties [43]. They demonstrate the hypolipidemic activities of MT-α-glucan and hypolipidemic properties, as well as an influence on the immunological function of diabetic mice.

### 3.6. Pulmonarius pleurotus (Grey Oyster Mushroom)

This kind of mushroom is a good source of anti-diabetic nutrients. It is high in polysaccharides, vitamins, and minerals, as well as dietary fibres, and is a good source of vital amino acids. Due to their excellent nutritional content and flavour, oyster mushrooms are eaten globally. They can be grown at a wide range of temperatures and have a plethora of health benefits [44].

### 3.7. Panellus serotinus (Mukitake)

It is Japan’s most tasteful edible mushroom. This mushroom has been discovered to be very helpful for the liver and aids in the prevention of non-alcoholic fatty liver disease [45].

### 3.8. Auricularia auricular-judae (Jew’s Ear/Black Fungus)

*Auricularia auricular*-judae are farmed extensively in China, Taiwan, Indonesia, Malaysia, Thailand, and the Philippines [46]. Agaricus, Pleurotus, Lentinula, Auricularia, and Flammulina. The majority of *Auricularia* species are edible and have therapeutic benefits. *Auricula polytricha* (Woodear mushrooms) and *A. auricula*-judae (black fungus) have antitumor, anticoagulant, and cholesterol-lowering properties. Additionally, *A. auricula-judae* exhibited hypoglycemic properties [45] through enzymes including laccase, superoxide dismutase, glucose oxidase, and peroxidase; polysaccharides including D-glucans and glucuronoxylomannan; lectins, proteins, glycoproteins, polysaccharide-protein complexes, and lipopolysaccharides; metal chelating agents, alkaloids, sterols, terpenoids, and phenolic compounds [42]. These constituents were found to target a variety of aberrant carbohydrate metabolic pathways, resulting in a reduction in blood glucose and insulin levels in type 2 diabetics. Efforts are now being made to quantify these chemicals in order to determine their therapeutic use in diabetes and other diseases. We presented our findings based on the hypoglycemic chemicals found in a variety of edible mushroom species. After taking into account the possible bioactive substances, in vivo and in vitro research was performed, and the results are shown in Table 1.

Because edible mushrooms have a high concentration of many bioactive and nutraceutical components, their bioactive components may be able to help with a wide range of health problems. In the treatment of a variety of lifestyle disorders, such as cancer, diabetes, liver disease, and cardiovascular disease, these are thought to be quite beneficial [66]. In addition to this, the fact that they contain antioxidants and antimicrobial compounds makes it possible for them to have anti-aging, immune-modulatory, and antimicrobial effects.

## 4. In Vivo Preclinical Study

Natural substance drug therapy is increasingly being seen as a viable alternative to standard diabetic care. This is particularly true in long-term conditions when insulin production is inadequate or where insulin is not used properly. The genus Reishi is very promising concerning its anti-diabetic activity. The most active components of these mushrooms are polysaccharides and triterpenoids [36]. The polysaccharide isolated from the hot fruiting body *G. lucidum* is made up of these sugars in the following molar ratios: 0.793% rhamnose, 0.964% xylose, 2.944% fructose, 0.167% galactose, 0.3840% mannose, and 7.94% glucose. β-Glycosidic bonding holds it together. The powder, which was hazel in color, could be dissolved in water [67,68,69,70,71]. Zhang and Lin were able to determine the hypoglycemic impact of *G. lucidum* polysaccharides after giving single doses of 25, 50, and 100 mg/kg of aqueous *G. lucidum* extracts to fasted mice. At 3 and 6 h after extract administration, blood glucose levels decreased according to the dosage. As early as 1 h after delivery, Gl-PS at 100 mg/kg raised circulating insulin levels by stimulating Ca^2+^ influx into pancreatic-cells [67,72,73]. A further polysaccharide, composed of glucose, mannose, xylose, arabinose, galactose, and ribose in the following ratio: 68.45:15.87:4.71:4.08:3.78:3.11, was isolated from *G. lucidum* in 2012 [69,74]. For 7 days, Xiao studied the effects of *G. lucidum* polysaccharides at 50 and 100 mg/kg/day in mice with streptozotocin-induced diabetes. Serum glucose, insulin, and white adipose tissue in the epididymis all dropped dramatically throughout the fast. Lower amounts of glycogen phosphorylase (EC 2.4.1.1), fructose-1, 6-bisphosphatase (EC 3.1.3.11), phosphoenol-pyruvate carboxykinase (EC 4.1.1.49), and glucose-6-phosphatase (EC 3.1.3.9, G6Pase) mRNA were also seen in the liver [75].

Seto et al. [76] studied the effects of *G. lucidum* extract by feeding it to mice at 0.03 and 0.3 g of food per kg of body weight for 4 weeks. Phosphoenol-pyruvatecarboxy kinase levels, which are typically elevated in diabetic mice, were considerably lowered after the administration of the extract [67,76]. Glucose synthesis in the liver was decreased and hyperglycemia was prevented because Gl-PS also inhibits glycogen synthase (EC 2.4.1.11) [67].

Due to free radical capture and suppression of NF-KB activity, Gl-PS protects against alloxane-induced pancreatic islet damage in vitro and in vivo [69]. Low-molecular-weight polysaccharides have been demonstrated to have hypoglycemic effects via influencing the mRNA expression of Bax, iNOS, and Casp-3 and to affect the metabolism of an anti-apoptotic protein (Bcl-2) and the expression of PDX-1. Thus, they prevent pancreatic cells from undergoing apoptosis and set in motion the regenerative process inside the body [42,77,78].

The next β-heteropolysaccharide, F31, with a weight-average molecular weight of 15.9 kDa, was isolated from Gl-PS in 2017 [79]. F31 may work by lowering insulin resistance and increasing the epididymal fat-to-bandwidth ratio by activating AMP-activated protein kinase (EC 2.7.11.31, AMPK) [72,79,80,81]. By decreasing lymphocyte infiltration, enhancing insulin-mediated insulin sensing in beta cells, and reducing plasma glucose in lean diabetic mice, LZ-8 may play a significant role in type 2 diabetes. The immunomodulatory effects of LZ-8 have been well-established, and it is now generally accepted that it may be used to manage diabetes [42,78].

Hikino et al. [82] isolated ganoderan A (23 kDa) and ganoderan B (7.4 kDa) from *G. lucidum*; both of these peptidoglycans had hypoglycemic action. The fungus’ mycelia and spore pericarp both contribute to the production of these chemicals [72,82]. In their study, they showed that ganoderan B reduced blood sugar levels in alloxan-induced diabetic rats. In addition, this chemical was shown to elevate plasma insulin levels in both normal and glucose-induced mouse models. Hepatic glucose-6-phosphatase and glycogen synthase activities were lowered, resulting in a lower liver glycogen content. Glucose-6-phosphate dehydrogenase (G6PDH, EC 1.1.1.49) and glucose-6-phosphate isomerase (EC 2.7.1.11) activities were dramatically elevated [82]. In 1986, ganoderan C (5.8 kDa), a third compound of the same kind, was discovered, and it also exhibited hypoglycemic action in mice with type 1 diabetes [67,83].

FYGL, another macromolecular proteoglycan, was purified using Sephadex G75 column chromatography after being extracted with ethanol and then water [84]. With a molecular weight of 72.9 kDa, FYGL was made up of 0.08 mol of arabinose, 0.21 mol of galactose, 0.24 mol of rhamnose, and 0.47 mol of glucose [69]. It caused a dose-dependent rise in blood insulin levels and blocked the activity of protein tyrosine phosphatase 1B (PTP1B) in skeletal muscle cells. Researchers have shown that this substance helps reduce blood sugar and cholesterol levels [75,84].

Wound healing issues are a frequent and important consequence of diabetes, and they need attention in any discussion of the disease. The possibility of limb loss increases. Several experiments were undertaken in 2012 to test the hypothesis that the polysaccharides in *G. lucidum* might promote and speed up the healing process of wounds. In mice with diabetes caused by streptozotocin (STZ), adding polysaccharide enhanced the incidence of scarring by around 21 percentage. Furthermore, polysaccharides from *G. lucidum* also partly activated wound angiogenesis and reduced oxidative stress in the mitochondria by inhibiting manganese superoxide dismutase (MnSOD) activity and nitration, glutathione peroxidase (GPx) activity, and phosphorylation of the redox enzyme p66Shc [68].

Very little is known about the additive effects of *G. lucidum* polysaccharides. Liu et al. [85] conducted research on rats with HFD/STZ-induced type 2 diabetes. A mixture of inulin and polysaccharides extracted from *G. lucidum*, when taken orally, dramatically improved body weight and balanced food consumption by enhancing carbohydrate utilization. This occurred as a result of improved insulin sensitivity. Diabetic rats were likewise shown to have lower levels of glycogen in their bodies. Glycogen levels in the liver and skeletal muscle are a useful indirect measure of insulin action; therefore, this is noteworthy. The data obtained indicate that the synergistic effect of inulin and *G. lucidum* polysaccharides may increase gluconeogenesis and hence control glucose levels [85].

One of the insulin signaling routes is the PI3K/Akt pathway. The kinase class phosphoinositide 3-kinase (PI3K) is crucial to the metabolic effects of insulin. Therefore, glucose and lipid metabolism may be affected by PI3K failure. Protein kinase B (Akt), a directly active PI3K molecule, is required for insulin to carry out its numerous biological functions. Phosphorylated Akt may improve glucose absorption into cells by increasing the translocation of glucose transporter type 4 (GLUT4) to the plasma membrane. In addition, it may influence glycogen production by inhibiting the phosphorylation of glycogen synthase kinase 3β (GSK3). The combination of *G. lucidum* polysaccharides and inulin improved insulin sensitivity in diabetic rats, as shown by an up-regulation of PI3K/Akt pathway gene expression and protein synthesis and an increase in Akt phosphorylation compared to the inulin group [85].

The impacts of *Agaricus blazei* on the pancreas of rats with streptozotocin diabetes were studied by Niwa et al. [86]. After two months of daily oral feeding of powdered mushroom biomass, histological analysis of the islets of Langerhans revealed an increase in the mass of pancreatic β-cells [86].

Another study looked at the effects of a glycoprotein preparation from *Agaricus bisporus* on mice that had 70% of their pancreas removed. The medication triggered pancreatic cell regeneration, indicating that inducing islet-cell proliferation had a therapeutic benefit [87]. This study also examined how aqueous extracts of *A. bisporus* biomass affected rats with a streptozotocin-induced diabetes paradigm. Raising the cellularity of the pancreatic islets of Langerhans and their apparent colonization by β-cells had an unanticipated impact in addition to lowering hyperglycemia and considerably increasing insulin levels [88].

Sirtuins are a kind of histone deacetylase that regulates cellular metabolism and psychological adjustment, as well as cellular longevity [89,90]. Zhou et al. and D’Onofrio et al. [91,92] found that sirtuin 1 regulates eNOS, resulting in less hyperglycemia-induced endothelial dysfunction. Furthermore, sirtuin 6 loss causes an increase in the expression of the pro-inflammatory cytokines IL-1 and IL-6 [93]. EGT may be an efficient regulator of cellular oxidative stress, inflammation, and survival pathways in vascular cells, according to recent investigations in cultured EC and isolated small vessels. In rat PC12 cells, a neuronal cell model, EGT protected against protein carbonylation, ROS generation, and cell death caused by glucose plus methylglyoxal. Furthermore, EGT inhibited the expression of nuclear NF-B and lowered the levels of AGE and its receptor (RAGE), limiting mitochondrial apoptosis [94]. However, investigations in streptozotocin-induced diabetic pregnant mice found that EGT defended against the development of neural tube abnormalities, reducing the prevalence of embryo deformities. There were no variations in maternal plasma glucose levels; these benefits were attributed to its antioxidant properties [95].

**Figure 4:**
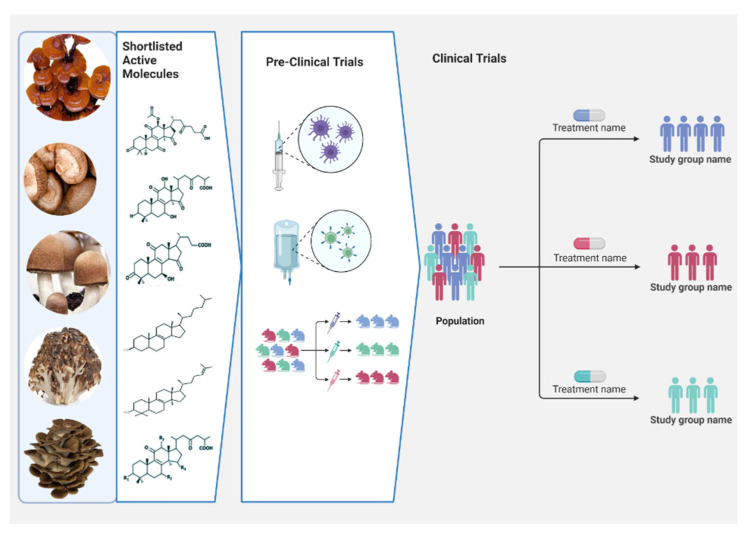
Overview illustration of preclinical and clinical evaluation of edible mushrooms to be used as therapeutics to prevent and treat the diseases.

Exopolysaccharides produced in the submerged culture of *Tremella fuciformis* in ob/ob mice were shown to have anti-diabetic properties [96]. Rushita et al. [97] investigated the hypoglycemic properties of a methanolic extract from *Pleurotus citrinopileatus* in rats with *streptozotocin-*induced type II diabetes. In comparison to the control group, those given a high dose of mushroom extract saw decreases in fasting blood glucose and serum catalase activity and increases in serum insulin. Mushrooms are a rich source of the polysaccharide glucan. It has been shown to restore pancreatic tissue function by increasing insulin production by β-cells, resulting in lowered blood glucose levels. Lectins isolated from *Agaricus campestris* and *A. bisporus* stimulated insulin release from islets of Langerhans in rat pancreatic tissues [98]. *Pleurotus ostreatus* ethanolic extract significantly decreased the blood glucose level in alloxan-induced diabetic mice. The blood levels of creatinine and urea were considerably lower in the treated groups. *P. ostreatus* has been shown to be beneficial in the production of therapeutic formulations for diabetes mellitus [99]. An overview of in vivo preclinical studies of the mushroom extracts is presented in Figure 4, and some mushroom polysaccharides with in vivo preclinical anti-diabetic potential and their brief mechanism of action are provided in the Supplementary File (Table S1, Supplementary Materials).

## 5. Clinical Significance of Mushrooms

Mushrooms have a long history of usage in Eastern medicine, which dates back to antiquity. In more recent times, mushrooms have been used for medicinal purposes in the modern world. The use of mushrooms and components derived from mushrooms in the management and cure of chronic illnesses has received a lot of attention in Westernized nations. This can be attributed to the growing interest in diets that are based more on plants than animal products. The highest concentration of the modified amino acid ergothioneine can be found in various types of edible mushrooms. It has been discovered that this substance can accumulate in almost all of the cells and tissues in the body, but it does so most frequently in cells and tissues that have been damaged by oxidative stress. [100]. The growing popularity of plant-based diets in Westernized nations has increased public awareness of the significance of mushrooms in the prevention and treatment of chronic illnesses [101].

Because obesity and food play such important roles in the development and management of type 2 diabetes, incorporating L-ergothioneine (EGT)-rich mushrooms into one’s diet, along with healthy eating habits, may be beneficial for people at risk of or with type 2 diabetes. The white button mushroom is widely farmed throughout Europe and North America, accounting for 35–45 percent of total global edible mushroom consumption [102]. According to retrospective research, consuming a typical serving of white button mushrooms (100 g, 3.2 mg EGT) daily for 16 weeks was associated with lower systemic oxidative stress and inflammation in people with pre-diabetes and two or more validated metabolic syndrome criteria. Serum EGT concentrations were increased 2-fold relative to baseline at the conclusion of the 16-week dietary intervention, which was related to a reduction in oxidative stress and inflammatory indicators. Advanced glycation end products (AGE) (carboxymethyl lysine, methylglyoxal derivatives) were reduced in blood, whereas oxygen radical absorbance capacity, a sign of antioxidant response, and adiponectin, an anti-inflammatory hormone, were increased [103].

EGT protected cells from high glucose-induced ROS generation, cell senescence, and decreased cell viability, according to D’Onofrio et al. [92]. Furthermore, studies of EC cytotoxicity in association with lower EGT levels in EC after high glucose exposure suggest that EGT may play a crucial role in EC cytotoxicity protection during hyperglycemia. Upregulation of sirtuins 1 and 6, which operate to downregulate the adapter protein p66Shc and the pro-inflammatory transcription factor NF-B, may be a mechanism by which EGT protects against glucose-induced EC senescence [92].

*G. lucidum* polysaccharides have been shown to have significant anti-diabetic potential in animal models; however, there is a lack of clinical evidence on their application in medicine. Polysaccharide fractions isolated from *G. lucidum* were included in a clinical investigation of ganopoly in 2004. It was administered to 71 persons with confirmed type 2 diabetes. Throughout the course of 12 weeks, they ingested 1800 mg of ganopoly three times a day. After 12 weeks of treatment with ganopoly, plasma glucose levels and glycosylated hemoglobin (HbA1C) levels decreased considerably [75,104]. Glucokinase, glucose-6-phosphatase, glucose-6-phosphate dehydrogenase, phosphoenol-pyruvate carboxykinase, fructose-1,6-bisphosphatase, phosphofructokinase, and glycogen synthase are all enzymes involved in the hepatic glucose metabolism pathway that is linked to the hypoglycemic effect of *G. lucidum* polysaccharides [42,78].

Monosaccharides, including glucose and fructose, are easily absorbed by the small intestine and transported into the bloodstream from digested dietary carbohydrates. The activity of β-glucosidase in the small intestine is related to oligosaccharide assimilation [105]. Several of *Genoderma*’s triterpenoids are potent inhibitors of this enzyme. Clinical trials of *Ganoderma* were conducted for a variety of illnesses, not only diabetes, because of its effective and potentially medicinal nature [38].

The impact of freeze-dried powders derived from the biomass of the edible fungi *P. ostreatus* and *Pleurotus cystidiosus* on healthy participants and persons with type 2 diabetes was studied. There was a decrease in blood glucose and a rise in insulin levels in diabetic individuals who took the medicine for a month. As per the study, the hypoglycemic effect is linked to an increase in glucokinase activity and insulin secretion stimulation, which results in higher glucose uptake by peripheral tissues and glycogen synthesis [106].

In Taiwan, a clinical experiment was carried out with 72 diabetic patients to see how well the mushroom *Agaricus blazei* worked as an anti-diabetic. Patients were also given gliclazide and metformin, plus a capsule with 500 mg of *A. blazei* three times a day. After 12 weeks, patients in this trial showed a significant reduction in insulin resistance, as measured by a drop in HOMAI IR scores [107]. Oral administration of *P. ostreatus* and *P. cystidiosus* at a dose of 50 mg/kg/body weight has been shown to increase serum insulin levels and decrease postprandial serum glucose levels in patients with type 2 diabetes [106]. Japanese type 2 diabetes patients were studied based on their dietary patterns, and the results showed that those who regularly ate mushrooms required less medication and led healthier lives [108]. From January 2009 to September 2010, the Bangladesh Institute of Research and Rehabilitation in Diabetes, Endocrine, and Metabolic Disorders (BIRDEM) ran a clinical trial with 200 g of *P. ostreatus* given to 73 diabetic housewives for a prolonged time of a year and found a significant lowering of glucose, total cholesterol, low-density lipoproteins, and blood pressure without negatively impacting the liver, kidney, or hemopoietic tissues [109]. Both *G. lucidum* and *Ophiocordyceps sinensis* are listed as among the safest drugs in the American Herbal Products Association’s Botanical Safety Handbook. Although there is a dearth of research on the optimal *Ganoderma* dosage, traditional Chinese medical practitioners have advocated for daily doses of 1.5–9 g of dried *Ganoderma* extract [38]. An overview of human clinical studies of the mushroom extracts is depicted in Figure 4. 

## 6. The Preventive Mechanistic Approach of Mushrooms against Diabetes and Insulin Resistance

Polysaccharides, terpenoids, and vitamin D have a major role in controlling diabetes through many distinct mechanisms. A few mechanistic examples relevant to insulin resistance are the blood glucose-lowering effect, inhibition of glucose absorption, and maintenance of pancreatic ß cell activity [110], which is briefly described.

### 6.1. The Polysaccharide-Mediated Blood Glucose-Lowering Effect

A glycosidic linkage connects simple sugars or monosaccharides to form polysaccharides, a widespread biopolymer. Homopolysaccharides are straight or highly branched and made up of the same monosaccharide molecules as another homopolysaccharide, while heteropolysaccharides are made up of distinct monosaccharide units [111]. Mushrooms have been found to have high concentrations of β-D-glucans, especially β-glucan, a form of dietary fiber that has shown positive effects in combating type 2 diabetes [112]. Gene expression of glycogen synthase kinase (GSK-3 β), glycogen synthase (GS), and glucose transporter 4 (GLUT4) in the liver and muscle is regulated by mushroom extracts from *Pleurotus* species [49], *Agaricus bisporus* [54], *Ganoderma lucidum* [113], etc., leading to decreased blood glucose levels. As a result, GS kinase 3 beta (GSK-3 β) could be a negative regulator of GS mediated by insulin [114]. Polysaccharides prevent insulin resistance through multiple mechanisms, including down-regulating α-amylase and α-glucosidase activities and promoting PI3K/AKT pathways, which play a crucial role in maintaining normal glucose levels [110].

### 6.2. Pancreatic β Cell Activity Maintenance

Polysaccharides (β-D-glucan) found in mushrooms have been shown to have powerful immunological modulatory effects, including the suppression of NF-kB activity and the elimination of oxidative damage. Mushroom polysaccharides and other bioactive substances protect beta cells in the pancreas from apoptosis and reduce glucotoxicity [112]. Extracts of the mushrooms *Pleurotus* spp., *A. bisporus*, etc., have been shown to have a considerable impact on β-cell functionality and hence maintain β-cell proliferation [57].

### 6.3. Glucose Absorption Inhibition

Mushrooms contain water-soluble dietary fiber, which slows digestion and reduces the postprandial glucose spike [115,116]. Multiple studies have shown that mushrooms, particularly *Pleurotus* spp. [49], *A. bisporus* [54], and *G. lucidum* [59], significantly reduce blood glucose levels by delaying the body’s absorption of glucose [110].

### 6.4. Terpenoid-Mediated Blood Glucose-Lowering Effect

Blood sugar rises as enzymes such as α-glucosidase and α-amylase break down oligosaccharides into simpler sugars [117]. Terpenoids [118] found in mushrooms (*Pleurotus* spp., *G. lucidum*, etc.) are thought to have an α-glucosidase inhibitory activity, which prevents the formation of monosaccharide molecules and facilitates glycogen formation in the liver and muscle.

### 6.5. Mushroom-Based Vitamin D in Blood Glucose Regulations

Mushrooms are members of the fungi kingdom and, unlike plants, contain a lot of sterol ergosterol in their cell walls. Ergosterol in the mushroom cell wall is converted to pre-vitamin D2 and then thermally isomerized to ergocalciferol, often known as vitamin D2, when exposed to sunlight [119]. Vitamin D, namely 1, 25-dihydroxyvitamin D, or 1, 5(OH) 2D, is crucial for maintaining healthy glucose levels. By acting both directly on β-cells and indirectly on other immune cells such as inflammatory macrophages, dendritic cells, and different types of T cells, it protects β-cells from damaging immunological responses [120]. Despite the availability of evidence, scientists are still unclear on the topic of vitamin D’s bioavailability in the treatment of diabetes [121]. However, new evidence from a randomized placebo-controlled experiment by Urbain et al. [122] demonstrates that vitamin D2 bioavailability in humans can be increased by eating UV-B-treated button mushrooms and that this effect is statistically indistinguishable from that of vitamin D2 supplementation [122].

## 7. Mushrooms as an Anti-Diabetic Functional Food

Mushrooms are nutrient-dense filamentous fungi with fruiting bodies that are high in protein and carbohydrates. They are also rich in minerals such as phosphorus, magnesium, selenium, copper, and potassium, as well as vitamins such as vitamin B and vitamin D and key amino acids that the body needs to operate properly [45,123]. Mushrooms have been used in human diets for a long time due to their possible health benefits, including their antibacterial, antioxidant, antiviral, anticancerous, and hypocholesterolemic effects [124]. Many types of mushrooms occur in nature, but only a handful are utilized and grown as foods [124]. Since high blood glucose levels are a sign of diabetes, diabetics must eat a healthy diet that helps them control their blood glucose. Despite their differences in look and flavor, all mushrooms share similar nutritional profiles that include minimal sugar and fat content, as well as high levels of minerals such as selenium and various vitamins B.

**Figure 5:**
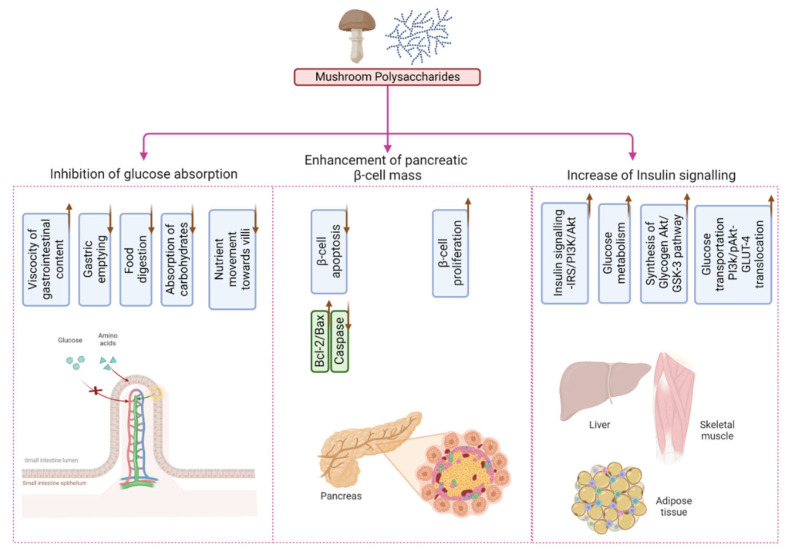
Involvement of mushroom polysaccharides in glucose homeostasis across multiple tissues and their anti-hyperglycemic 1effects Inhibition of glucose absorption; Enhancement of pancreatic β-cell mass; Increase in insulin signaling).

They may be regarded as a great dietary option for diabetic individuals (Figure 5) because of their low calorie content and low glycemic index. Mushrooms have therapeutic qualities because they contain a variety of secondary metabolites, including polysaccharides, alkaloids, antibiotics, lectins, lactones, terpenoids, and metal-chelating agents [125]. These secondary metabolites are bioactive molecules with high therapeutic potential. Bioactive compounds were traditionally derived primarily from field-cultivated mushrooms. This manufacturing method was and continues to be time-consuming and labor-intensive, with little control over the product’s quality and output of desired metabolites [126]. Although most mushroom components can be used medicinally, mycelia have far greater bioactivity than fruiting bodies and spores. Therefore, submerged mushroom mycelia cultivation is a promising technique for the effective large-scale synthesis of mycelia biomass and value-added secondary metabolites in a condensed space, in a shorter amount of time, and with less contamination [127]. Bioactive metabolites can be affected by a number of factors, including environmental conditions (oxygen concentration, pH, temperature, incubation time, etc.), medium composition (carbon source, nitrogen source, types of salts, special additives such as vegetative oils, and vitamins), and fermentation mode and methodology (agitated culture or static culture) [128]. The primary goal of optimizing culturing conditions is to accelerate mycelia development and increase secondary metabolite synthesis, specifically polysaccharides and triterpenoids, which are the most active components in mushrooms. [129]. This technique is applicable in practice because it can be used to generate the highly efficient production of numerous secondary metabolites by addressing the primary issues that affect the fermentation process and purification systems. Following a review of the available research on the anti-hyperglycemic effects of isolated compounds and extracts obtained from various mushroom species, it is possible to conclude that two types of chemicals are particularly important: terpenoids and polysaccharides.

As edible mushrooms have low cholesterol, fat, and carbohydrate content while being high in protein, mineral, and vitamin content, they are considered low-calorie meals for diabetics [130]. Mushrooms have been characterized as the finest sources of natural pharmaceuticals with anti-diabetic properties by Kaur et al. and Chaturvedi et al. [131,132]. These are recognized functional foods and are a substantial source of bioactive chemicals such as proteins, lipids, and polysaccharides, as well as metabolites with high therapeutic activity such as alkaloids, lectins, sterols, terpenoids, and phenolic components.

## 8. Challenges

For the significant establishment of mushroom-based prevention and treatment of diabetes, more research in the clinical field is needed, such as in vivo animal studies, in vitro enzyme inhibition assays (amylase, glucosidase, pancreatic lipase, and DPP4-dipeptidyl peptidase 4), human trials, pilot studies, and prospective and retrospective studies. The link between vitamin D and insulin resistance, as well as enzymatic assays and their potential influence, must be given special attention. Furthermore, without appropriate inquiry, reaching a judgment is difficult. As a result, clinical investigations should be expanded [116].

Furthermore, these mushroom-based medications are completely natural and inexpensive, making them accessible to the general public. Furthermore, many herbal therapies currently in use have not received a thorough scientific evaluation, and some of them have the potential to induce serious adverse consequences and major drug–drug interactions [133].

Specific criteria and precise recommendations for their use in hyperglycemia are, however, absent; this is likely owing to a lack of data on the efficacy of mushroom species on diabetes and a lack of a clear molecular mechanism to ensure their anti-diabetic potential. As a result, research into the precise molecular mechanism that can reveal their anti-diabetic potential is required. Animal studies have provided the most conclusive proof of medicinal mushrooms’ therapeutic properties [27].

More controlled human trials are needed, especially for long-term use. Long-term safety concerns about mushroom consumption, as well as their interactions with other medications, necessitate further explanation. As a result, further research into the aforementioned difficulties is required to justify the use of mushrooms and their compounds as potential medications or nutraceuticals for the management of diabetes. According to the data, there are still many locations where the variety and range have yet to be discovered, and new taxa could have physiologically viable metabolites with therapeutic activity for diabetes management. As a result, much mushroom study is needed, particularly for any natural substance that should always be converted into its appropriate dosage forms, such as tablets, capsules, or pellets, from a commercial standpoint. However, this section for mushrooms has received little attention. As a result, it is critical to look into the possibility of turning these mushrooms’ extracts into therapeutic goods. Of course, this would entail overcoming the obstacles connected with their formulation development so that they can be recognized as nutraceuticals and reach patients with diabetes and other metabolic illnesses at the bedside. The many studies and discussions on mushroom polysaccharides described and suggested that antioxidant-active mushroom polysaccharides should be explored further and that their development as therapeutic agents in the treatment of diabetes should be supported [134].

## 9. Future Prospects and Outlook

Bioactive compounds found in mushrooms have been shown to have a wide range of medicinal and pharmacological effects in animal and human models in in vitro and in vivo investigations. Other dietary fibers and saccharides help to prevent hypertension, hyperlipidemia, diabetes, and immunomodulatory disorders. The terpenoids and phenolic chemicals found in the mushroom protect the heart, liver, neurons, kidneys, and liver. India has 300–315 edible mushroom species (Basidiomycetes) and 357 genera worldwide, but only 39 mushroom species have been studied for their anti-diabetic properties. Bioprospecting novel taxa for anti-diabetic efficacy and biomolecule isolation requires further research. Lead bioactive compounds in clinical trials and research on cytotoxicity and drug action can help produce noble therapies from mushrooms to better the lifestyles of millions of people worldwide [135]. To tackle the concerns and obstacles associated with mushroom plantations, future mushroom research opportunities could include the development of new growing technology and post-harvest processing procedures. In addition, more research investigations, including complete human clinical trials, are needed to better understand the mechanism and metabolic pathways of mushroom bioactive interactions and create useful data. To fully use the potential of mushrooms for the benefit of human health and life, more extensive research on unexplored wild edible variations and their production conditions is required.

Mushroom polysaccharides (β-glucans) restore pancreatic tissue function, increasing β-cell insulin production and reducing blood glucose. Medicinal mushrooms provide endless possibilities for developing new diabetic treatments. Nevertheless, several glucose metabolism systems have complex signaling pathways. The mechanism of action of these bioactive compounds needs more research and clinical studies.

In addition to nutritional and physiological benefits, mushroom growing has broader implications in terms of poverty alleviation, livelihood and employment prospects, environmental protection, and so on. The unique properties and biological functions of the different bioactives found in mushrooms (particularly polysaccharides, proteins, and antioxidant chemicals) have sparked a strong interest in their cultivation. Because these varieties thrive in naturalistic environments, local communities should be included in their commercial cultivation. To achieve equal economic growth and socioeconomic effects through mushroom production, appropriate regulations and processes must be implemented. To accomplish effective cultivation of mushrooms, research studies on the effect of growing circumstances, substrate content, and harvest timing on the phytochemical content and nutritional value of mushrooms must be carried out in detail. In addition, further research is needed to completely comprehend the mechanism and metabolic pathways by which mushroom bioactives exert their pharmacological effects. Supplementary drugs are becoming increasingly popular among patients to manage their distress during and after treatment, in addition to standard medicines. There is little information on the cost and benefits of pharmacologic and non-pharmacologic treatments that are used as medicinal mushrooms.

## 10. Conclusions

There have been a number of studies on new ways to prevent diabetes or its complications, but it is still not clear how they can be used in real life. The potential medicinal benefits of edible mushrooms have been recognized for centuries, making them a fascinating target for the development of novel therapeutics. There are many cultures around the world that revere mushrooms for their medicinal and nutritional properties, and mushrooms are widely consumed as a result. Several pathophysiological pathways linked to the development of diabetes have been shown to be regulated by biologically active metabolites and components derived from edible mushrooms, demonstrating their glucose-controlling effects. Edible mushrooms have been studied for their potential to reduce hyperglycemia by looking into their antioxidant defenses, carbohydrate metabolism pathways, α-glucosidase and aldose reductase inhibitory activities, β-cell enhancement, and insulin-releasing activity. Potential therapeutic applications of mushrooms warrant further investigation, including pre-clinical and clinical studies, enzyme inhibition assays, human trials, pilot studies, and prospective and retrospective studies. Vitamin D and insulin resistance, considering its potential effect through an enzymatic assay, also require special attention. Furthermore, it is difficult to draw a conclusion without conducting extensive research. This is why it is crucial for clinical research to uncover more information. Based on the evidence we have, we can say that mushrooms are helpful and have a lot of potential to treat noncommunicable diseases such as diabetes.

## Figures and Tables

**Table 1:** Bioactive ingredients of edible mushrooms and their medicinal effect.

S. No.	Name of the Species	Secondary Metabolites	Bioactivity	References
1	*Calvatia gigantea*	2-Pyrrolidinone, 1-Dodecene, ergosterol, hexadecane, benzeneacetic acid	Anti-diabetic, antioxidant, anti-inflammatory	[47]
2	*Coprinus comatus*	Mycelium, polysaccharides	Immunomodulatory, anti-diabetic, antioxidant, anti-cancer	[48]
3	*Pleurotusostreatus*, *P. pulmonarius*, and *P. fossulatus*	Terpenoids, heterocyclic amines, phenols, glucan, proteoglycan	Anti-cholesterol, anti-cancer effects, anti-inflammatory, anti-diabetic	[49,50]
4	*Boletus edulis*	Tocopherol, quinic acid, hydroxy benzoic acid	Antioxidant, anti-inflammatory, hypoglycemic	[51]
5	*Grifola frondosa*	Grifolan polysaccharide, D-fraction, MD-fraction, polysaccharide, galactomannan, heteroglycan	Hypoglycemic, anti-inflammatory, anti-modulatory, anti-tumor	[52,53]
6	*Agaricus bisporus*	Pyrogallol, hydroxybenzoic acid derivatives glavonoid	Anti-inflammatory, anti-diabetic	[54,55]
7	*Morchella esculenta*	Polysaccharides (mannose, galactose, and glucose), phenolic compounds	Antioxidant, anti-inflammation, immunoregulation, hypoglycemic	[56]
8	*Hericium erinaceus*	4-chloro-3, 5-dimethoxybenzoic acid-*O*-arabitol ester, 2-hydroxymethyl-5-α-hydroxyethyl-γ-pyranone, 6-methyl-2,5-dihydroxymethyl-γ-pyranone, 4-chloro-3,5-dihydroxybenzaldehyde, 4-chloro-3,5-dihydroxybenzyl alcohol	Immunomodulatory, hypoglycemic, antimicrobial	[57,58]
9	*Ganoderma lucidium*	Ganoderic acid, danoderiol, danderenic acid, lucidenic acid, *Ganoderma leucidum* Polysaccharide	Anti-diabetic, anti-inflammatory	[53,59]
10	*Lenzites betulina*	α-glucan, β-glucan, β-glucan protein, galacturonic acid	Antioxidant, anti-hyperglycaemic, anti-inflammatory, anti-proliferative, antibacterial	[60]
11	*Flammulina velutipes*	Flammulinolide, enokipodin, proflamin and other polysaccharide	Anti-tumor, anti-hypertension, antihypercholesterolemia, hypoglycemic	[61,62]
12	*Lentinula edodes*	Lentinan, eritadenina	Anti-carcinogenic, antioxidant, hypocholesterolemic action	[63,64]
13	*Termitomyces robustus*	glutamyl-βphenylethylamine, tryptophan 1,4-hydroxyphenylacetic acid, hydroxyphenyl propionic acid and phenyllactic acid	Hypoglycemic effect	[65]

## Data Availability

The data that support the findings of this study are available from the corresponding author upon reasonable request.

## Supplementary Materials

The following supporting information can be downloaded at: https://www.mdpi.com/article/10.3390/molecules28062837/s1, Table S1: Mushroom Polysaccharides with in vivo preclinical anti-diabetic potential. References [136,137,138,139,140,141,142,143,144,145,146,147,148,149,150,151,152,153,154,155,156] are cited in the supplementary materials.
