# Supplementary material for: Exploring Edible Mushrooms for Diabetes: Unveiling Their Role in Prevention and Treatment
Source: Molecules. 2023 Mar 21;28(6):2837. doi: 10.3390/molecules28062837 (PMC10058372; doi:10.3390/molecules28062837)
Supplement: Supplementary file 1 [file molecules-28-02837-s001.zip › molecules-2129109-supplementary.pdf]

Table S1. Mushroom Polysaccharides with *in vivo* preclinical anti-diabetic potential.

Sl. No.	Mushrooms	Polysaccharides	Mechanisms	References
1	*Agaricus blazei*	β-glucan	Treatment of diabetic mice with 20 mg ml^{-1} for 5 weeks decreased body weight and increased insulin production from the pancreas compared to the control group.	[136]
2	*Agrocybe chaixingu*	Polysaccharide	Prevent synthetical generated DNA sequences by sodium nitroprusside. Boost pancreatic cell protection from STZ-induced death in mice.	[137]
3	*Auricularia auricula-judae*	Water soluble polysaccharide	Reduce blood glucose and urine glucose and increasing hepatic glycogen. Reduced body fat, blood sugar, serum insulin, and HbA.	[138]
4	*Catathelasma ventricosum*	Selenium polysaccharide	Protecting the liver, kidneys, and pancreas from the harmful effects of diabetes and exibits hypo-lipidemic action in diabetic rats.	[139]
5	*Cordyceps militaris*	Pyran ring polysaccharide with α and β configuration.	Enhance insulin sensitivity and preserve the organs in type 2 diabetics.	[139]
6	*Cordyceps militaris and Ophiocordyceps sinensis*	Polysaccharide	Blood glucose levels in STZ-induced diabetic mice were reduced by 60%-70%.	[140]
7	*Grifola frondoas*	Polysaccharide. Glucose, mannose, galactose, xylose, arabinose, ribose, arabinose, xylose.	Lowers blood glucose level BUN, Scr, NAG in serum and high albumin in urine of diabetic rats, expression of P-NF kBp65 and P-IkBα in the kidneys. Reduce inflammation and improve insulin production in diabetic rats. Maintain a healthy amount of insulin and glucose in the blood when fasting. Target PI3-AKT pathway.	[141,142]
8	*Lentinus edodes*	Exopolymer	Improve plasma insulin level, repair damage of pancreatic β cells, upregulation of insulin synthesis, and lowers plasma glucose (21.5 %), plasma cholesterol (25.1 %), and triglycerides (44.5 %).	[143]
9	*Phellinus linteus*	α linked glycosyl residues	Reduces blood glucose levels and blocks apoptosis made by H_2O_2 in diabetic mice.	[144]
10	*P. linteus*	Polysaccharide enriched powder	Fasting blood glucose levels in diabetic rats were significantly lowered in vivo experiments at doses of 300 and 600 mg/kg body weight for 8 weeks.	[145]
11	*Pleurotus abalonus*	[→6)-α-D-Glcp-(1→] n.	Polysaccharide treatment reduces blood glucose levels in diabetic mice.	[146]
12	*P. citrinopileatus*	Water soluble polysaccharide	Diabetic mice exhibited a decrease in fasting blood glucose.	[147]
13	*P. djamor*	Zinc polysaccharide	Increase in SOD, GSH-PX & CAT followed by, diminished levels of MDA, ALT, AST, BUN, CRE, TC, LDL-C & HDL-C in liver, kidney, and serum of STZ-induced diabetic mice. Decrease of blood glucose in mice as to control one.	[148]
14	*P. eryngii*	Exo-polysaccharide	Exhibits inhibiting properties in mice (α-amylase, α-glucosidase). Eliminating both EPS 1 and EPS 2 lowers blood sugar. Suppress the high level of ALB, BUN, CRE, & UA. Protect kidney damage.	[149]
15	*P. florida*	Polysaccharide	Streptozotocin-induced diabetic mice treated with polysaccharides at 200 and 400 mg/kg showed a decrease in blood glucose, serum cholesterol, triglyceride, urine glucose, and ketones, as well as a restoration of superoxide dismutase, catalase, and reduced glutathione.	[150]
16	*P. ostreatus*	Polysaccharide	Decreases levels of hyperglycemia and hyperlipidaemia. GSK3 phosphorylation and GLUT 4 protein expression increased.	[151]
17	*P. sajor caju*	Glucan rich polysaccharide, β-glucan rich polysaccharide	Up-regulate GLUT 4, adiponectin and down regulate the expression of IL-6, TNF-α, SAA2, CRP & MCP 1 via reduction of NF-KB transcription factor.<br>Turn on the AMPK unit in 3T3-L1 cells, which regulates glucose and lipid metabolism.	[152,153]
18	*P. tuber regium*	Extracellular polysaccharide	Up- regulate PPAR-α mRNA, protein levels which further down regulate obesity, hyperglycemia, hyperlipidemia and progression of diabetes.	[154]
19	*Tremella fuciformis*	Exo-polysaccharides	Improve insulin sensitivity and lower plasma glucose by 52% compared to control mice may through regulation of PPAR- Y.	[155]
20	*T. mesenterica*	Acidic polysaccharide Glucuronoxylo mannan	Blood glucose levels are reduced by glucuronoxylomannan treatment in diabetic rats caused by streptozocin alone or by STZ plus nicotinamide.	[156]
